# A case of left atrial free ball thrombus in a patient with prosthetic mitral valve

**DOI:** 10.21542/gcsp.2021.8

**Published:** 2021-04-30

**Authors:** Ahmed Mohamed Fawzy Ellaien

**Affiliations:** Department of Cardiology, Faculty of Medicine, Menoufia University, Menoufia, Egypt

## Abstract

A free left atrial ball thrombus is relatively a rare diagnosis and usually leads to systemic embolization or fatal complication of thrombus incarceration in left ventricle inflow. We report a case of 52-year-old woman who was admitted to hospital with symptoms of heart failure. Routine echocardiography showed a free-floating ball thrombus in the left atrium. The free-floating thrombus was round with smooth surface and about 37 mm in diameter. The mitral valve prosthesis was well-functioning. The computed tomography of the patient also showed the left atrial ball thrombus. The erratic finding was that the patient was compliant on oral anticoagulants for the last 6 months with her INR in theraputic range. This suggested that this thrombus had been there for a some time and anticoagulants failed to resolve this problem. She was referred to cardiac surgery for thrombus extraction but the patient refused to have the operation.

## Introduction

The incidence of free-floating ball thrombus in the left atrium is quiet rare^1^ and usually occurs in dilated left atrium on top of mitral stenosis, or after mitral valve replacement^2^. This case report includes the clinical and echocardiographic signs of a free ball thrombus discovered in the left atrium in a patient who had undergone mitral valve replacement nine years previously. Free-floating ball thrombus usually leads to catastrophic complications of thrombus incarceration in left ventricle inflow and sudden death^3^. The presence of free-floating thrombus also leads to higher embolic potential^4^.

Anticoagulation and thrombolytic therapy seem to have no role in the immediate management of left atrial free thrombi and surgical extraction remains the first choice treatment ^5^, however anticoagulation long term role in prevention of thrombus recurrence is evident.^4^

## Case Report

A 52-year-old woman, who had a bi-leaflet mechanical prosthesis placed in the mitral position 9 years previously, was admitted to hospital because of paroxysmal dysnea, orthopnea, generalized edema and easy fatigability for 2 weeks. She had never before experienced embolic manifestation or syncopal attacks. She was maintained on a regimen of adequate anticoagulation with warfarin (12 mg). The international normalized ratio was 3.6 on day of admission.

**Figure 1.**
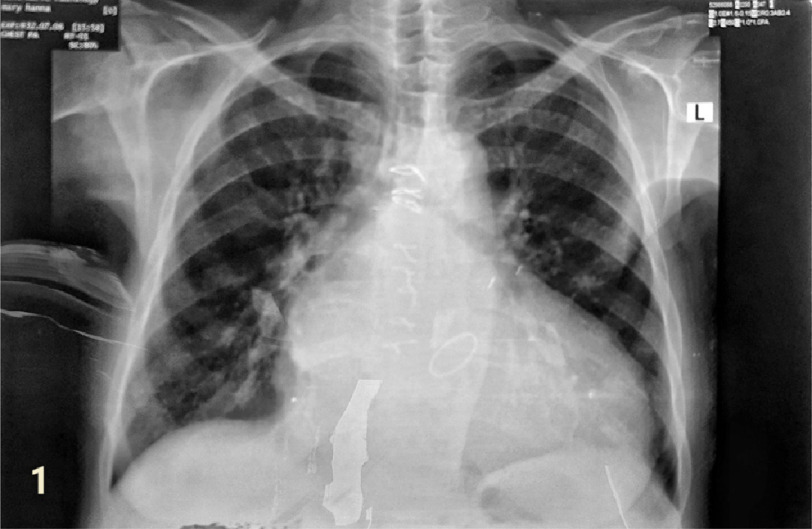
Chest x-ray showing enlarged LA, biventricular enlargement, and pulmonary venous congestion.

At time of hospital admission, she was in New York Heart Association (NYHA) class 4 and in atrial fibrillation (AF) with rapid ventricular response. Her blood pressure was 100/70 mmHg. She was given furosemide 40 mg IV to relieve pulmonary congestion and digoxin 0.5 mg IV to control the heart rate. On auscultation, the prosthetic valve click was normal, bilateral medium-sized crepitations were heard over the chest wall. An ECG revealed AF with rapid ventricular rate of 120 b/m and poor R progression in the chest leads. The chest radiograph showed cardiomegaly, left-atrial enlargement, biventricular enlargement, and pulmonary venous congestion (Figure 1). Transthoracic echocardiography revealed well-functioning mitral valve prosthesis. The left atrium was markedly dilated (72 mm) with existence of a large, rounded mobile thrombus 37 mm in diameter freely floating in the left atrium (Figures 2a and 2b).

**Figure 2a.**
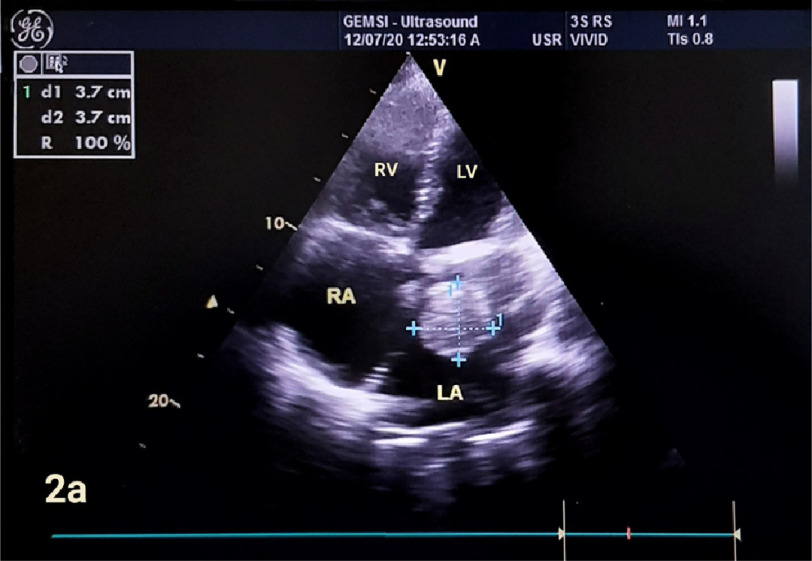
Transthoracic echocardiography. Apical 4-chamber view showed large freely mobile ball thrombus 37 mm in diameter floating in dilated LA. LA =left atrium, LV =left ventricle, RA =right atrium, RV =right ventricle.

**Figure 2b.**
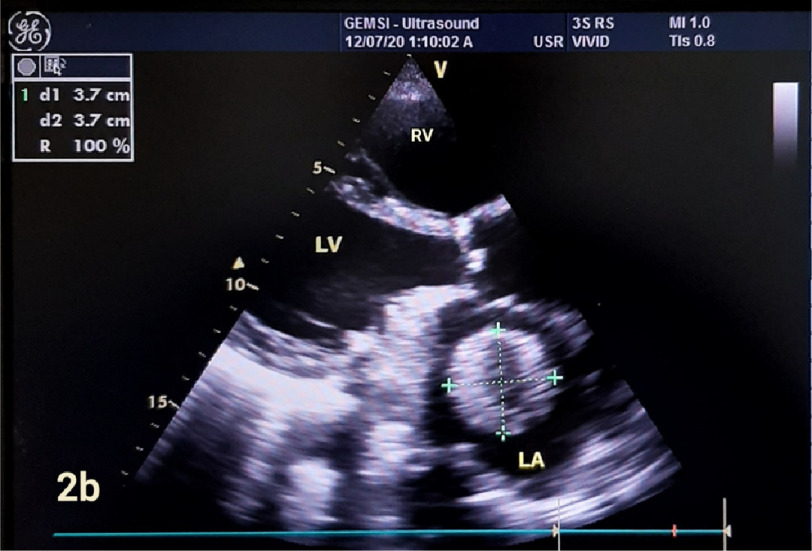
Parasternal long axis view, showing the same ball thrombus with the same dimensions.

The relatively large size of the thrombus, compared to mitral prosthesis orifice, seemed to protect the patient from systemic embolization of the thrombus. Computed tomography of the chest also revealed the left atrial ball thrombus (Figures 3a and 3b). The patient was referred to cardiac surgery for thrombus extraction, but she refused the operation despite full disclosure of the potentially fatal consequence of her case.

**Figure 3a.**
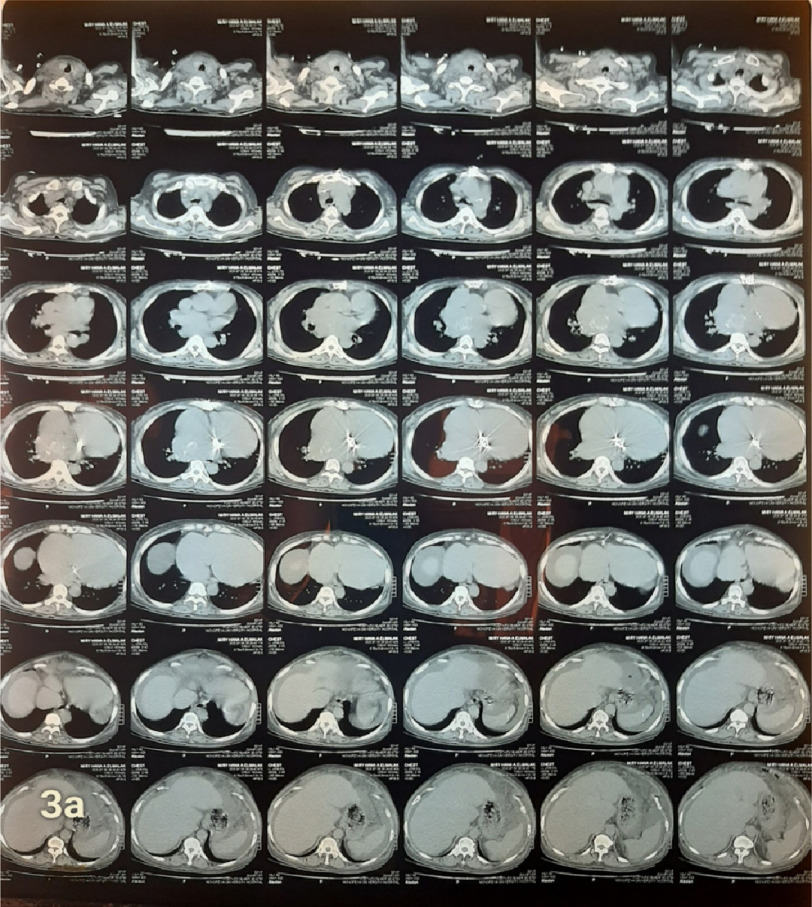
Computed tomography of the patient showing dilated cardiac chambers and round mass in the LA.

**Figure 3b.**
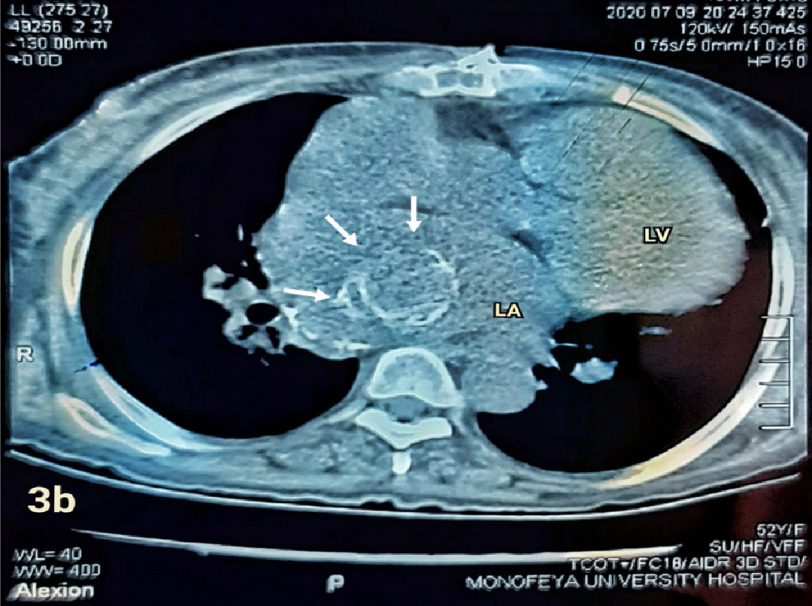
Computed tomography of the patient showed dilated cardiac chambers and round mass in the LA.

## Discussion

Thrombus formation in the left atrium can occur as either an early or late complication of mitral valve replacement, but the presence of ball thrombus, freely floating in the left atrium without attachment to the mitral valve prosthesis or the atrial wall, is very rare^1^. Although rare, early detection & management is important because of its potentially fatal complications ^5,6^.

Ball thrombus usually originates as a mural thrombus adherent to the interatrial septum and to a lesser extent to atrial appendages^2^. Mural thrombus slowly increases in size to become a projecting mass connected to the atrial wall through a pedicle. The bulbous part of the thrombus becomes more bulky, with progressive deposition of fibrin and platelets, and the pedicle thins out and finally detaches or breaks down into multiple small fragments, causing systemic embolization events. Ball thrombus freely rotates in the dilated left atrium with a characteristic smooth fine surface^6^.

The reason for the typical spherical or oval shape of free ball thrombi remains unclear. It has been suggested that this configuration comes from fresh clot being layered out concentrically as it spins around the atrium^1,2^.

The movement of the thrombus results in a ping-pong effect characterized by sudden acceleration due to the impact of the mitral leaflets during ventricular systole. A floating rotatory movement within the left atrial cavity, and a rotation of the thrombus on its own axis that does not follow the cardiac cycle, are highly suggestive of a ball-valve thrombus^1^ .

Clinical diagnosis is difficult, but LA free thrombus should be suspected if patients with mitral stenosis, or mitral valve prosthesis, and atrial fibrillation have intermittent or changing murmurs, embolic manifestations and syncope. Variations in symptoms have also been associated with changes in the position of the patient^6^. Our patient demonstrated none of these features, and an echocardiogram was necessary to reveal the presence of a free-floating thrombus. The diagnosis of a free floating thrombus in echocardiography is based on two criteria: the thrombus must be bigger than the cross sectional area of the valve orifice, so that it is locked up within the atrium, and it should have a smooth surface with no attachment to the atrial wall^7^.

Diagnosis of a free-floating ball thrombus necessitates emergency surgery with simultaneous treatment of the underlying cause. Surgery produces a long-term survival rate of more than 90%^1^. Anticoagulant and thrombolytic therapy do not appear to play a role in the acute management of left atrial ball thrombus^3,5^.

## Data availability

The datasets used during the current study are available from the corresponding author on reasonable request.

## Conflict of interest

The author reports no conflicts of interest.

## Funding

This research did not receive any specific grant from funding agencies in the public, commercial, or not-for-profit sectors.
